# Polymyxin resistance in Gram-negative bacteria: a comprehensive review with a focus on the role of 4-amino-4-doexy-l-arabinose

**DOI:** 10.1007/s11033-025-11368-4

**Published:** 2025-12-29

**Authors:** Wanesa Maria Sasal, Dawid Gmiter, Wiesław Kaca

**Affiliations:** 1 Department of Microbiology, Institute of Biology, Faculty of Natural Sciences, Jan Kochanowski University of Kielce, Kielce, Poland; 2 Institute of Microbiology, Faculty of Biology, University of Warsaw, Warsaw, Poland

**Keywords:** 4-amino-4-deoxy-l-arabinose, ArnT transferase, Resistance mechanisms, Gram-negative bacteria, Polymyxins

## Abstract

Polymyxin resistance among Gram-negative bacteria poses a serious global health threat by limiting treatment options for multidrug-resistant (MDR) infections. This review summarizes current knowledge on the molecular mechanisms underlying polymyxin resistance, with a particular focus on the role of 4-amino-4-deoxy-l-arabinose (l-Ara4N) in lipopolysaccharide (LPS) modification. We discuss the regulation of l-Ara4N biosynthesis and transfer by two-component systems such as PmrAB, PhoPQ, CrrAB, CprRS, ColRS, and ParRS, which mediate bacterial responses to environmental stimuli. Furthermore, we synthesize recent findings on combination therapies designed to restore polymyxin efficacy, including agents such as natural polyphenols, antimicrobial peptides, and secondary metabolites. Special attention is given to emerging strategies targeting ArnT, including inhibitors with ent-beyerane skeletons (e.g., BBN149 from *Fabiana densa var. ramulosa*). Finally, this review highlights ongoing challenges related to polymyxin toxicity and underscores the need for future research aimed at optimizing dosing strategies and reducing adverse effects. By integrating findings across multiple studies, this review provides an updated overview of current approaches to counteract polymyxin resistance in Gram-negative bacteria.

## Introduction

The discovery of antibiotics revolutionized medicine. However, over the past several decades, resistance to commercially available antibiotics has increased significantly among pathogenic bacterial strains [1]. This issue, known as antimicrobial resistance (AMR), poses a serious global threat to human health [2]. The gradual increase in resistance began in the 1970s and has since evolved into a worldwide concern, officially recognized by the World Health Organization (WHO) [3, 4]. The greatest challenge is posed by the so-called “ESKAPE” pathogens: *Enterococcus faecium*, *Staphylococcus aureus*,* Klebsiella pneumoniae*,* Acinetobacter baumannii*,* Pseudomonas aeruginosa*, and *Enterobacter* spp. The term refers to a group of microorganisms capable of evading the effects of currently available antimicrobial agents through a variety of resistance mechanisms [5]. These pathogens are also categorized as multidrug-resistant (MDR), extensively drug-resistant (XDR), or pandrug-resistant (PDR) strains and are widespread globally. However, they do not represent the full spectrum of MDR pathogens. Other clinically relevant species, such as *Escherichia coli*,* Salmonella enterica*,* Neisseria gonorrhoeae*, and *Mycobacterium tuberculosis*, also pose serious therapeutic challenges and contribute significantly to the global AMR burden [6, 7]. Moreover, resistance continues to increase, largely due to intrinsic defense mechanisms and to the ability of bacteria to transfer antibiotic resistance genes among different species [4, 6].

Infections caused by MDR pathogens are associated with high mortality rates worldwide and have become a major public health problem of the 21st century. The phenomenon is primarily driven by the overuse of antibiotics and our limited capacity to develop new antibiotics. Initially, MDR bacteria were mainly associated with hospital-acquired infections. However, they have now spread beyond healthcare facilities and are considered one of the leading causes of community-acquired infections. The increasing incidence of MDR-related infections has led to increased mortality, higher healthcare costs, and the more intensive use of antibiotics [8, 9]. Polymyxins (PMs), including polymyxin B (PMB) and colistin (CST, polymyxin E), have been reintroduced for the treatment of infections caused by Gram-negative MDR bacteria due to their strong bactericidal activity [1, 10]. Although their use was previously limited by nephrotoxicity and neurotoxicity, novel formulations and dosing strategies now help reduce adverse effects. Intravenous administration of these antibiotics has increased, yet reports indicate a rise in heteroresistance in Gram-negative pathogens such as *K. pneumoniae*,* A. baumannii*, and *P. aeruginosa* [11]. This highlights the urgent need for greater understanding of their pharmacological properties in order to optimize their clinical applications and minimize the risk of resistance developing among pathogenic microorganisms [1]. In this review, we provide a structured synthesis of the current state of knowledge on the molecular mechanisms underlying PMs resistance and the regulatory networks controlling gene expression. We also highlight recent advances in combination therapy strategies aimed at enhancing the antimicrobial efficacy of PMs while mitigating their toxicity.

## Polymyxins as antibiotics of last resort

PMs are secondary peptide metabolites produced by the Gram-positive bacterium *Paenibacillus polymyxa*. They belong to an older class of nonribosomal cyclic lipopeptide antibiotics, first discovered in 1940s [12]. The first discovered polymyxin was polymyxin A (formerly known as ‘aerosporin’), which was isolated during the fermentation of *Bacillus aerosporus* (later renamed *Paenibacillus polymyxa*). To date, five types of PMs have been identified (polymyxins A, B, C, D, and E), but only two, PMB and CST (polymyxin E), have been used in clinical practice [13, 14]. In 1959, CST was introduced to clinical use for the treatment of infections caused by Gram-negative bacteria. However, by the early 1970s, the use of both PMB and CST was largely discontinued because of their significant neurotoxicity and nephrotoxicity [12]. On July 26, 2023, the WHO published an updated classification of antibiotics within the Access, Watch, and Reserve (AWaRe) system. According to this classification, PMB is included in the Reserve group, which comprises antibiotics of last resort, used to treat infections caused by MDR pathogens [15].

### Chemical structure of polymyxin B and colistin

**Fig. 1:**
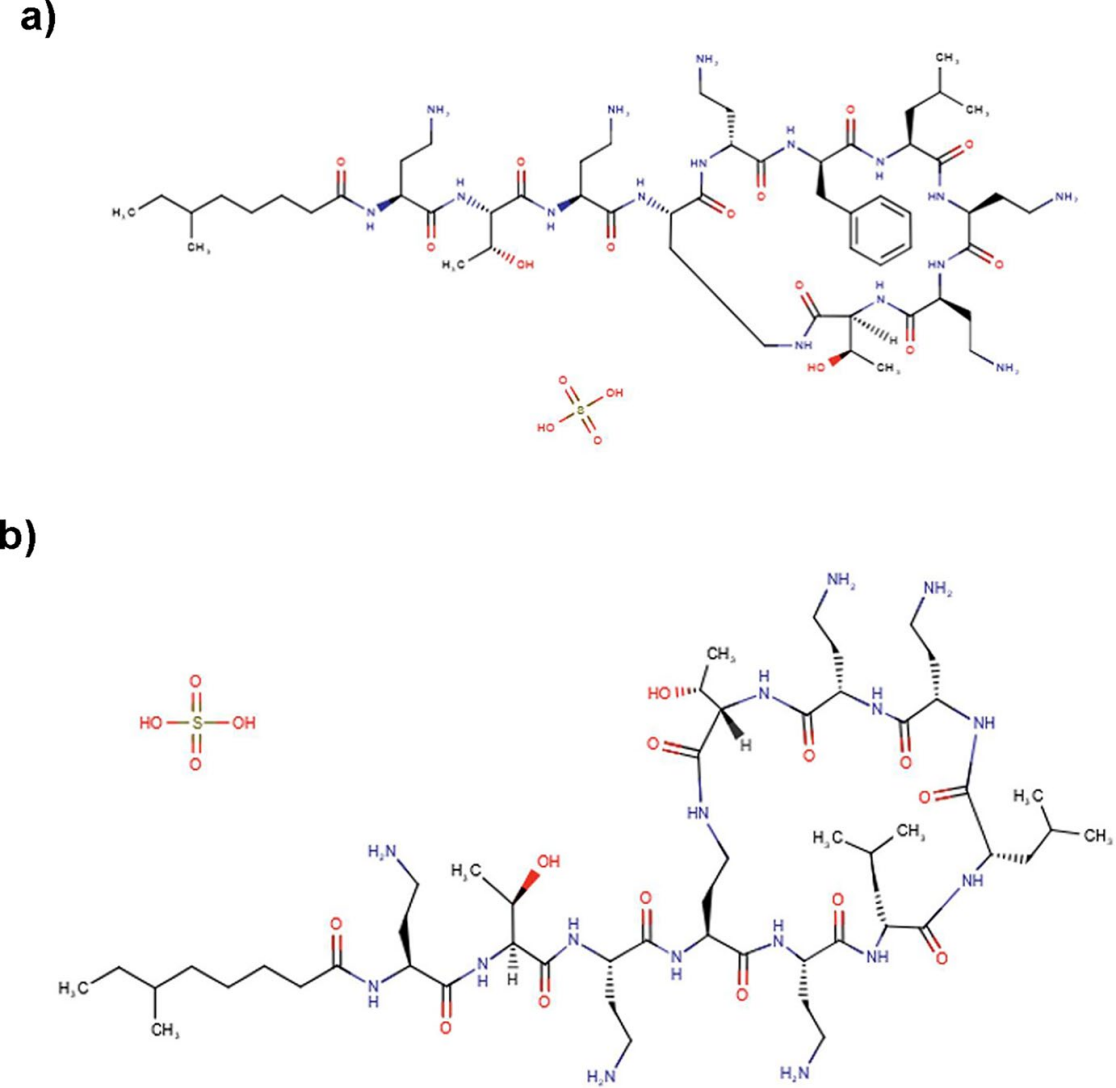
Chemical structure of polymyxin B sulfonate (**a**) and colistin sulfate (**b**) [DrugBank https://go.drugbank.com/]

The core structure of PMB and CST comprises a cyclic heptapeptide linked to a tripeptide side chain, acylated at the N-terminus by a fatty acid **(**Fig. 1**)** [12, 16]. A single amino acid substitution distinguishes them: d-phenylalanine occupies position 6 in PMB, whereas d-leucine occurs at the same position in CST [11, 17]. Due to this structural similarity, both compounds display nearly identical antimicrobial spectra and mechanisms of action [18]. A key distinction between them lies in their routes of administration: PMB is administered parenterally as a sulfate salt, whereas CST is administered as colistin methanesulfonate (CMS), an inactive sodium salt prodrug. CMS undergoes hydrolysis both in vitro and in vivo to release the active form of CST [1, 19].

### Synthesis of polymyxin B and colistin

**Fig. 2:**
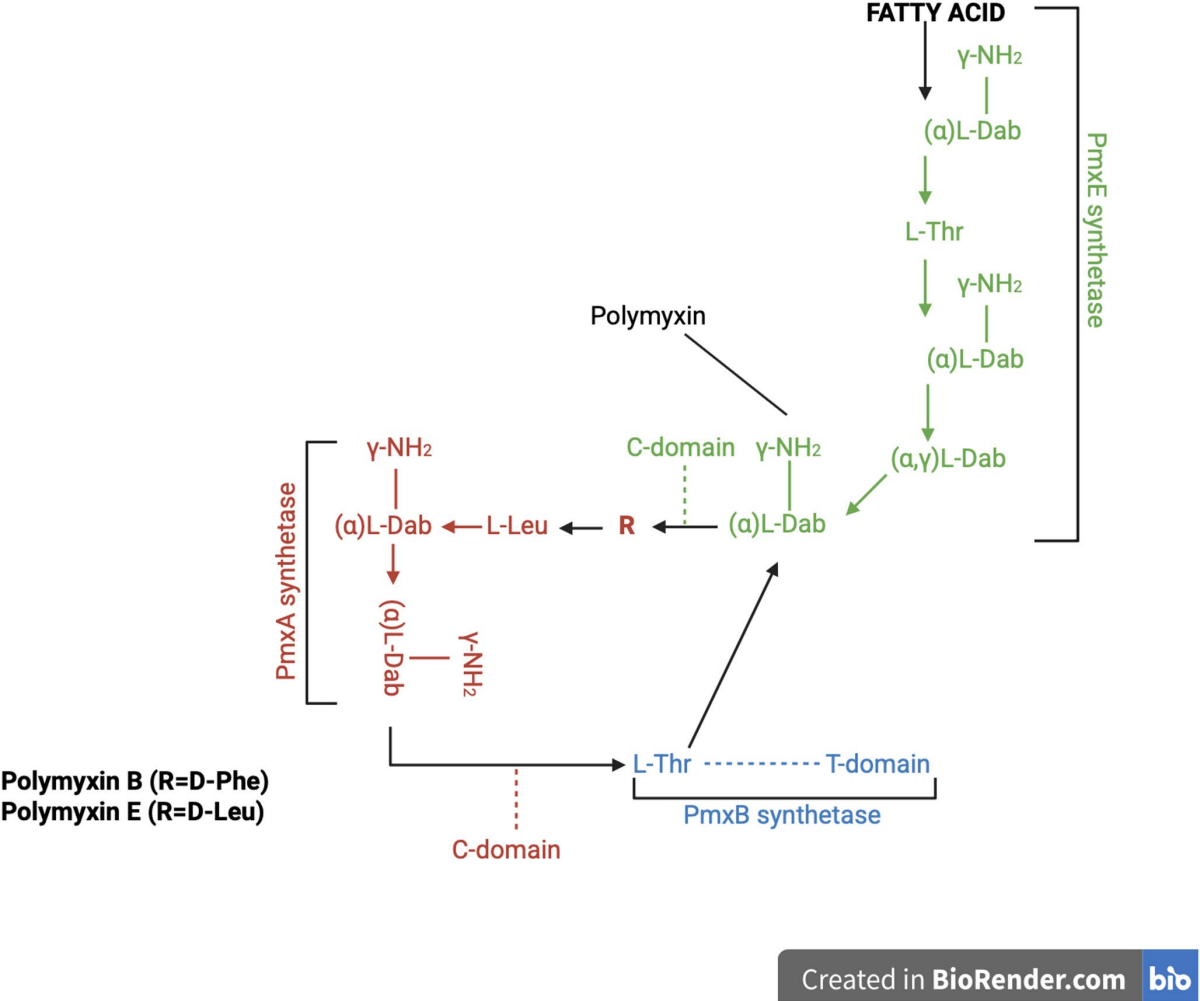
Biosynthetic pathway of PMB and CST in *Paenibacillus polymyxa*. PMs are synthesized by three nonribosomal peptide synthetases (NRPSs), PmxA, PmxB, and PmxE, and exported by two membrane-associated transport proteins, PmxC and PmxD. The structure includes a fatty acid moiety (either 6-methyloctanoic acid or isooctanoic acid) and the amino acids threonine (Thr), phenylalanine (Phe) or leucine (Leu), and α,γ-diaminobutyric acid (Dab), with α and γ indicating the positions of the amino groups (–NH₂) involved in peptide bond formation [12]. Created in https://BioRender.com

The synthesis of PMs is mediated by the nonribosomal peptide synthetase (NRPS) system, which has three domains, responsible for adenylation (A), thiolation (T), and condensation (C). NRPS may also contain auxiliary modules, such as termination and epimerization domains [12, 20]. These domains and modules are typically encoded within a biosynthetic gene cluster. In the case of PMs, this is the biosynthetic *pmx* gene cluster, consisting of five open reading frames: *pmxA*, *pmxB*, *pmxC*, *pmxD*, and *pmxE*. The proteins PmxA, PmxB, and PmxE are PMs synthetases, whereas PmxC and PmxD are transmembrane proteins that play crucial roles in transporting both the precursor and the final product across the bacterial cell membrane Fig. 2 [12, 21].

### Antibacterial mechanism of polymyxins

Polymyxin antibiotics are considered agents of last resort for the treatment of infections caused by Gram-negative MDR bacteria [22]. These bacteria have a characteristic envelope composed of an inner membrane (IM) and an outer membrane (OM) [23]. The OM functions as a semipermeable barrier that facilitates the uptake of essential nutrients while restricting the entry of harmful substances, including antibiotics [22]. It is primarily composed of lipopolysaccharide (LPS), which is the main molecular target of PMs [17, 24]. LPS is structured into three distinct domains: the hydrophobic lipid A moiety, which anchors the molecule in the OM; a conserved oligosaccharide core; and the variable O-specific polysaccharide (O-antigen). The primary target of PMs is negatively charged lipid A. The phosphate groups within lipid A coordinate divalent cations, such as Mg²⁺ and Ca²⁺, forming magnesium and calcium bridges that stabilize the LPS structure. PMs have a threefold greater affinity for these phosphate groups than LPS, displacing the cations and thereby disrupting the organization of LPS and increasing the permeability of the OM [1, 22]. This destabilization facilitates the formation of membrane defects, allowing the influx of external compounds, including the antibiotic itself, which ultimately contributes to cell death [1]. PMs also perturb the integrity of the bacterial envelope by disrupting the interaction between the IM and OM, leading to significant changes in the membrane architecture. Although the exact mechanism of bacterial killing remains incompletely understood, studies have shown that despite membrane destabilization, there is no consistent evidence of cytoplasmic leakage or lysis under standard conditions [22].

### Spectrum of activity of polymyxins

The strong structural similarity of PMB and CST underlies their nearly identical mechanisms of action and similar antimicrobial efficacy [25, 26]. PMs are characterized by a narrow spectrum of activities and are primarily used in the treatment of infections caused by Gram-negative pathogens, particularly members of the family Enterobacteriaceae. These include *Enterobacter* spp., *Citrobacter* spp., *Escherichia coli*, *Shigella* spp., and *Salmonella* spp. Activity has also been observed against nonfermenting Gram-negative bacteria, including *Stenotrophomonas maltophilia*, *P. aeruginosa*, and *A. baumannii*. PMs lack activity against Gram-positive bacteria, anaerobic microorganisms, and Gram-negative cocci such as *Neisseria* spp. [27]. Furthermore, several species are intrinsically resistant to PM antibiotics, including: *Morganella morganii*, *Serratia marcescens*, *Chromobacterium* spp., *Legionella* spp., *Vibrio cholerae*, *P. mallei*, *Providencia* spp., and *Proteus* spp. [27, 28]. The predominant mechanism by which bacteria develop resistance to PMs involves alterations of the cell membrane structure that reduce interactions with these antibiotics. This is primarily achieved through modifications of the charge within the LPS component, leading to electrostatic repulsion of the cationic PMs molecules. Additional factors may also contribute to resistance, including active efflux, reduced membrane permeability, and capsule formation [29].

PMB has demonstrated potent activity against MDR bacteria, which pose a major threat to public health due to the global rise in antimicrobial resistance. According to the U.S. Centers for Disease Control and Prevention (CDC), critical MDR pathogens include carbapenem-resistant Enterobacterales, *P. aeruginosa*, and *A. baumannii*, all of which are listed among the top priority organisms for which new therapeutic strategies are urgently required [30].

### Pharmacodynamics and pharmacokinetics of polymyxins

There is still limited information on the pharmacodynamics and pharmacokinetics of polymyxin antibiotics, posing challenges for the establishment of optimal dosing regimens. The pharmacodynamic and pharmacokinetic profiles of antimicrobial agents are critical determinants in the optimization of dosing strategies, which is essential for effective patient treatment [31]. Landersdorfer et al. (2017) conducted murine studies demonstrating that the antibacterial activity of PMB against *K. pneumoniae* is best correlated with the ratio of the area under the unbound drug concentration time curve to the minimum inhibitory concentration (fAUC/MIC), a key PK/PD index [30, 32]. The PK/PD ratio is considered the most reliable predictor of the in vivo efficacy of both PMB and CST against bacterial pathogens [33].

The intravenous administration of CMS results in a progressive increase in plasma CST levels, because only 20%–25% of CMS is converted to its active form. Consequently, standard dosing regimens may require over 36 h to attain steady-state therapeutic plasma concentrations [34, 35]. For a 75 kg adult patient, a loading dose of 300 mg of CST base activity (equivalent to 9 million international units, IU) is recommended, followed by maintenance dosing of 150 mg every 12 h. This dosing regimen results in plasma CST concentrations of approximately 2 mg/L. Given the limited pulmonary penetration of CST, a plasma concentration of 2 mg/L is considered sufficient to treat lower respiratory tract infections, provided that the pathogen’s MIC does not exceed 1 mg/L [34, 36]. In contrast, CMS undergoes rapid conversion to CST in the urinary tract, leading to high intraluminal CST concentrations. Therefore, colistin is generally favored over PMB for the treatment of urinary tract infections [34].

PMB does not require conversion to an active form after intravenous administration, resulting in relatively consistent pharmacokinetics. Following typical dosing (loading dose 2.0–2.5 mg/kg; maintenance dose 1.25–1.5 mg/kg), serum peak concentrations (Cmax) reach approximately 2–14 mg/L, with a half life of 9–11.5 h [34, 37]. The drug is eliminated via both renal and non-renal pathways, with urinary recovery below 5%. Its pharmacokinetics are not significantly affected by renal function, allowing target steady state plasma concentrations to be achieved with standard dosing even in patients with creatinine clearance above 80 mL/min [34].

### Nephrotoxicity and neurotoxicity of polymyxins

Therapies involving PMs are associated with significant adverse effects, most notably nephrotoxicity and neurotoxicity. Despite these limitations, the clinical use of PMs has increased markedly in response to the global increase in MDR bacterial pathogens and the therapeutic challenges they pose [38]. Polymyxin neurotoxicity has been attributed to oxidative stress and mitochondrial dysfunction within neuronal cells. Preclinical studies in animal models suggest that mitochondrial dysfunction is mediated by PM uptake by specific receptors expressed on the neuronal membrane. These include peptide transporter 2 (PEPT2), the endocytic receptor megalin (MR), and organic cation transporter novel type 2 (OCTN2). This process leads to the generation of excessive reactive oxygen species (ROS), ultimately triggering the apoptosis of neural cells [38]. Nephrotoxicity is believed to result from direct toxic effects on renal tubular epithelial cells, particularly within the proximal tubules [38]. Clinical data indicate that nephrotoxicity is more common in patients receiving high dose CST than in those treated with PMB, and that CST toxicity is dose dependent, mostly mild to moderate, and reversible in most cases. Appropriate dosing of CST is therefore essential to minimize kidney injury. Additionally, advancing age and concomitant use of vasopressors have been identified as factors contributing to polymyxin associated nephrotoxicity. Taken together, these findings support that PMB has a potentially lower risk of nephrotoxicity than CST [34, 39, 40].

The neurotoxic effects of PMs are predominantly peripheral, given their relatively high molecular weight, which limits their effective penetration of the blood–brain barrier (BBB). However, some studies have reported that LPS derived from Gram-negative bacteria can disrupt the integrity of the BBB, enhancing the penetration of the central nervous system by PMs [41]. Polymyxin induced nephrotoxicity is a common adverse effect. In a meta-analysis including 95 cohorts (*n* = 7,911 patients), the pooled prevalence of nephrotoxicity was 26.7% (95% CI: 22.8–30.9%) for CST and 29.8% (95% CI: 23.8–36.7%) for PMB [42]. Additional analyses, including a cohort of 290 patients, reported an incidence of acute kidney injury (AKI) ranging from 40 to 45%, with a substantial proportion of cases being reversible upon discontinuation of therapy [43]. In comparison, a prospective study involving 317 courses of CST therapy reported that neurotoxicity was considerably less frequent (approximately 7%), whereas the incidence of nephrotoxicity was substantially higher, ranging from 20 to 60% for PMB; neurological manifestations included ataxia, dizziness, peripheral neuropathy, paresthesia, neuromuscular blockade, visual disturbances, disorientation, and seizures, all of which were reversible following discontinuation of PMs therapy [44].

## Lipid A modification and other mechanisms of resistance in Gram-negative bacteria

### Modifications of LPS structure

**Fig. 3:**
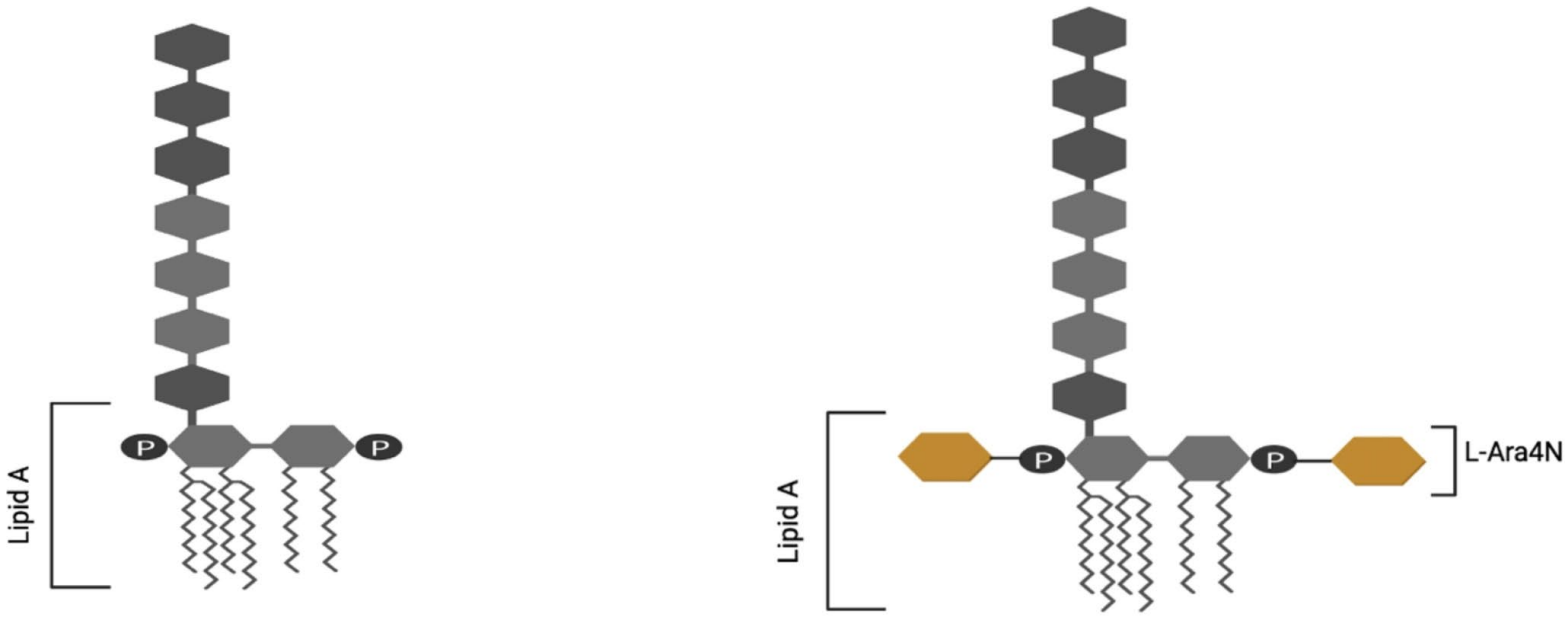
Simplified schematic of the LPS structure without the l-Ara4N moiety (left) and with the l-Ara4N moiety (highlighted in yellow) attached to lipid A (right). Created in https://BioRender.com

Gram-negative bacteria have developed multiple mechanisms of resistance to various antibiotics, including capsule formation, LPS loss, lipid A modification, the activation of efflux pumps, biofilm formation, target-site mutations, changes in the cell morphology, and the production of hydrolytic enzymes [45, 46, 47, 48, 49]. Table 1 provides a summary of Gram-negative bacterial species and their various antibiotic resistance mechanisms, conferring resistance to PMs. In this review, we focus specifically on the modification of the lipid A component of LPS by the addition of the positively charged 4-amino-4-deoxy- l-arabinose (l-Ara4N) moiety, and the regulatory factors governing this process. The incorporation of positively charged l-Ara4N (Fig.3) neutralizes the native negative charge on lipid A, thereby inhibiting the binding of cationic antimicrobial peptides, such as PMs [50]. The addition of l-Ara4N represents one of the two predominant structural modifications of LPS that can completely neutralize the net negative charge on lipid A. The second major modification involves the incorporation of phosphoethanolamine (PEtN), which reduces the net negative charge on lipid A from − 1.5 to − 1. Given its greater capacity to neutralize the lipid A charge, l-Ara4N modification is considered more effective than PEtN incorporation in conferring resistance to cationic antimicrobial agents [51].

**Table 1:** Examples of Gram-negative bacteria and their resistance mechanisms to polymyxins

Modifications	Examples of Gram-negative bacteria	Determinants / involved genes	References
Modifications in LPS structure (lipid A); reducing negative charge on LPS	*P. aeruginosa*, *A. baumannii*, *Salmonella enterica*,* Yersinia spp.*, *B. cenocepacia*, *Proteus mirabilis*	Addition of PEtN, l-Ara4N and/or galactosamine to lipid A of LPS. Activation of two-component systems (e.g., PhoPQ, PmrAB); presence of antimicrobial peptides	[22, 45, 50, 52, 53, 54]
Deacylation of lipid A (*Salmonella* only)	*Salmonella enterica*	Removal of (R)-3- hydroxybutyrate at position 3. Activation of *pgaL* by PhoPQ	[51]
mcr-mediated modification of lipid A	*E. coli*, *K. pneumoniae*,* Enterobacter spp.*,* Salmonella spp.*,	pEtN transferase (MCR-1) encoded by *mcr* genes (e.g., *mcr-1* to *mcr-10*)	[55, 56]
Loss of LPS	*A. baumannii*, *P. aeruginosa*, *N. meningitidis*, *M. catarrhalis*	Inactivation of genes involved in lipid A biosynthesis (*lpxA*, *lpxC*, *lpxD*)	[22, 52, 57]
Efflux pumps	*K. pneumoniae*, *A. baumanii,* *P. aeruginosa*	Efflux pump regulators: BrlR, Sap proteins, KpnEF, AcrAB-TolC, MexXY/OprM	[22, 52, 58]
Capsule formation	*K. pneumoniae*, *N. meningitidis*	Polysaccharide capsule formation; expression of cps operon in presence of PMB	[22, 59]

### Biosynthesis and attachment of l-Ara4N moiety to LPS

**Fig. 4:**
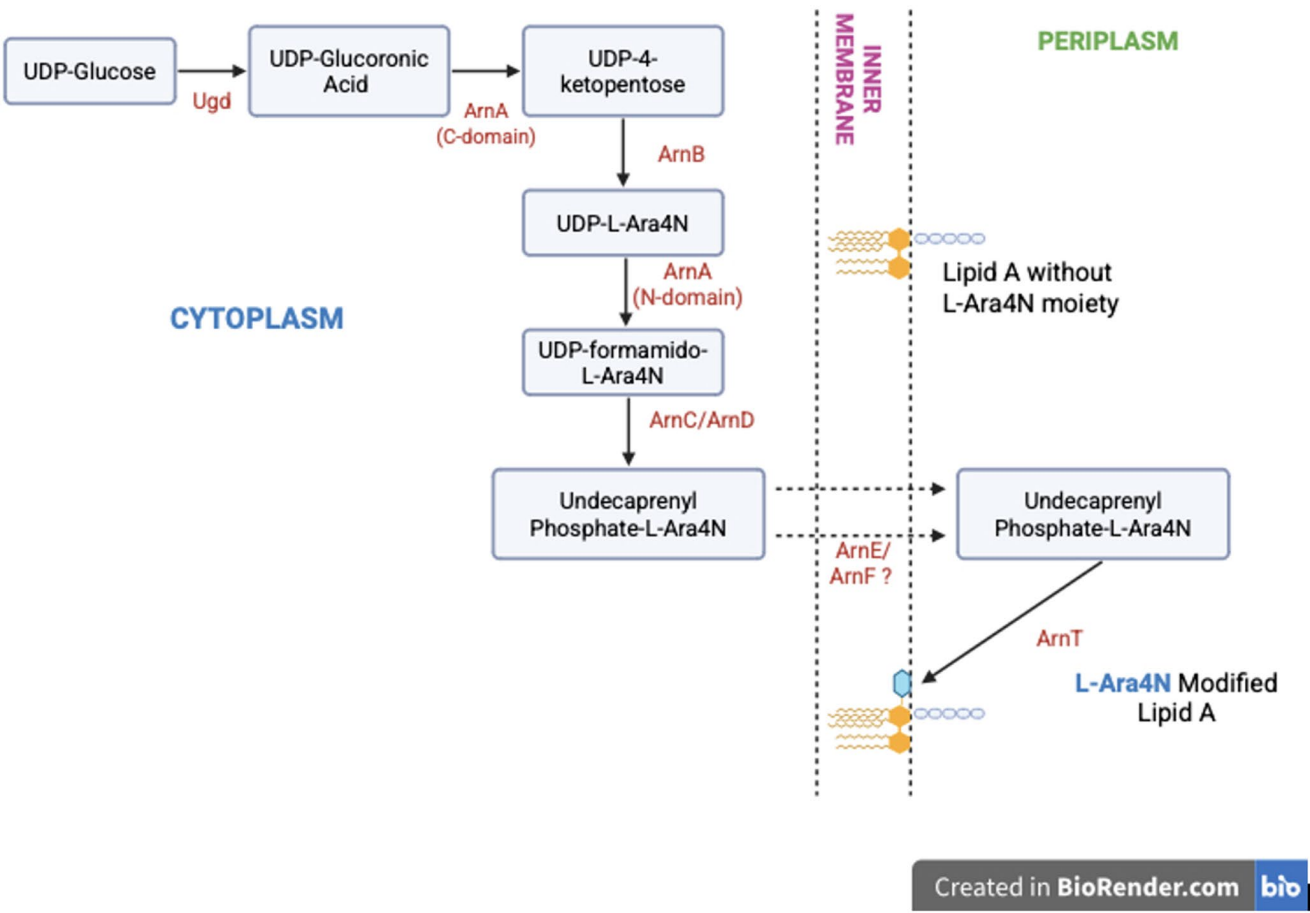
Biosynthesis of l-Ara4N and its incorporation into lipid A. The pathway begins with the oxidation of UDP-glucose to UDP-glucuronic acid. The C-terminal domain of ArnA then catalyzes an NAD⁺-dependent oxidative decarboxylation to generate UDP-4-ketopentose, which is subsequently converted to UDP-l-Ara4N by ArnB. The N-terminal domain of ArnA transfers a formyl group, yielding UDP-4-formamido-l-Ara4N. This nucleotide sugar is then transferred to undecaprenyl phosphate by ArnC and deformylated by ArnD to produce undecaprenyl-Phosphate-l-Ara4N. The lipid-linked intermediate is translocated across the inner membrane, most likely with the assistance of ArnE and ArnF. Finally, the membrane glycosyltransferase ArnT transfers l-Ara4N to the 4′-phosphate of lipid A in the periplasmic leaflet. This modification decreases the net negative charge of lipid A and contributes to resistance against cationic antimicrobial peptides such as polymyxins [44, 52]

The biosynthesis and attachment of the l-Ara4N moiety to the lipid A domain of LPS is mediated by the *arnBCADTEF* operon (also known as *pmrHFIJKLM*) and the *ugd* gene (*pmrE*). The initial step involves the enzyme ArnA (PmrI), the C-terminal domain of which catalyzes the oxidative decarboxylation of UDP-glucuronic acid, resulting in the formation of UDP-4-ketopentose [53, 60]. This intermediate is then transaminated by ArnB (PmrH) to produce UDP-l-Ara4N. The N-terminal domain of ArnA then catalyzes the formylation of UDP-l-Ara4N, a critical step because only the N-formylated derivative acts as a substrate for ArnC, which transfers the moiety to undecaprenyl phosphate. The formyl group is then removed by ArnD, yielding undecaprenyl phosphate conjugated with l-Ara4N. This lipid-linked intermediate is then translocated to the OM, where ArnT (PmrK) mediates the transfer of l-Ara4N to the 4′-phosphate group of lipid A (Fig. 4) [53, 61]. In a recent in silico analysis, the distribution and sequence conservation of the *arnT* gene component of the *arnBCADTEF* operon was examined across multiple *Proteus mirabilis* strains, the results revealed strong conservation [62].

**Fig. 5:**
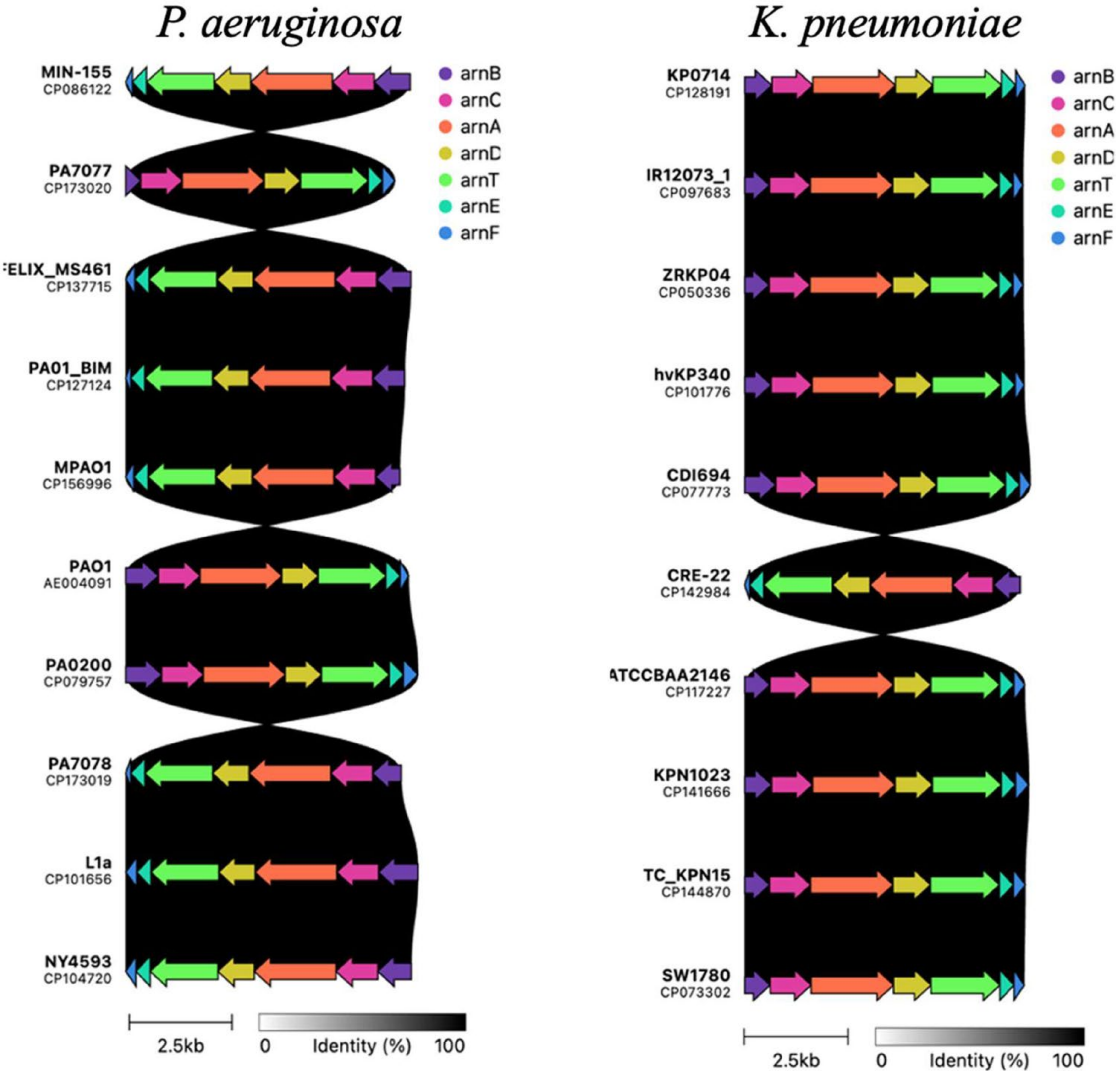
Distribution and diversity of *arn* genes among varios strains of *P. aeruginosa *(on the left) and *K.pneumoniae* (on the right). Genomic sequences avaiable in public databases of the National Center for Biotechnology Information (NCBI) were used for the analysis. Data visualization was performed using the Clinker software

A comparative genomic analysis of the *arnBCADTEF* genes in *P. aeruginosa* and *K. pneumoniae* strains (performed in this study) showed high similarity of these genes and encoded proteins with over 98% amino acid sequence identity, underscoring their evolutionary conservation among Gram-negative bacteria Fig. 5). Further supporting the importance of *arnT*, a deletion mutant study in *Salmonella Typhimurium* (JOL2943) demonstrated that loss of *arnT* leads to significantly reduced resistance to PMB, impaired intracellular survival, diminished biofilm and swarming capacity, and lower induction of cytokines such as IL-1β in host cells. These findings highlight key role of *arnT* in lipid A modification, immune invasion and virulence [63].

## Key Two-Component systems (TCSs) regulating the attachment of l-Ara4N

The biosynthesis and attachment of l -Ara4N to the LPS structure is tightly regulated by two-component regulatory systems (TCSs). Among the most extensively studied are the PmrAB and PhoPQ systems, which play central roles in resistance mechanisms not only by mediating lipid A modifications but also by exerting broader regulatory effects, including the modulation of efflux pump expression and membrane stress responses. Mutations within the components of these TCSs are frequently associated with acquired resistance to PMs [64, 65, 66, 67, 68, 69, 70]. Each TCS typically comprises a membrane-bound histidine kinase (HK) and a cytoplasmic response regulator (RR), which together mediate signal transduction in response to environmental stimuli. The signal transduction cascade proceeds via five principal steps: (1) the detection of the external stimulus by the HK sensor domain; (2) the autophosphorylation of HK on a conserved histidine residue; (3) the transfer of the phosphate group to a conserved aspartate residue on RR; (4) the transcriptional activation or repression of RR-regulated target genes; and (5) the dephosphorylation and inactivation of RR [45]. Although the regulatory pathways governing lipid A modification are often species specific, the PmrAB and PhoPQ systems are conserved and functionally active in several Gram-negative pathogens, including *A. baumannii*, *Salmonella enterica* serovar Typhimurium, *P. aeruginosa*, and *Yersinia* spp. [71].

### PhoPQ

The PhoPQ two-component system consists of two key proteins: PhoP, a DNA-binding response regulator, and PhoQ, a transmembrane sensor kinase. PhoQ detects acidic pH, low Mg²⁺, and cationic antimicrobial peptides (CAMPs), initiating autophosphorylation and subsequent phosphate transfer to PhoP. Activated PhoP regulates genes responsible for LPS modification, including the addition of l-Ara4N [72, 73]. PhoPQ influences the modification of LPS, including the attachment of l-Ara4N, both directly and indirectly. Indirect regulation occurs through the PmrD protein, which enhances the expression of the *arnBCADTEF* operon (*pmrHFIJKLM*) and the *ugd* (*pmrE*) gene by stabilizing phosphorylated PmrA through the inhibition of its dephosphorylation. The transcription of *pmrD* itself is activated by phosphorylated PhoP, establishing a regulatory link between the two major systems involved in PMs resistance PhoPQ and PmrAB. Amino acid substitutions leading to constitutive PhoP activation result in upregulated *pmrD* expression and increased resistance to PMs. Environmental conditions such as low pH, extracellular DNA, low Mg²⁺ concentrations, and the presence of CAMPs also promote *pmrD* transcription. A well characterized example of indirect PhoPQ-mediated l-Ara4N attachment via PmrD occurs in *S.enterica* [22, 74, 75].

Another factor influencing the attachment of l-Ara4N to the LPS structure is Ecr, a small transmembrane protein found in *Enterobacter* species. Ecr enhances the transcription of genes involved in l-Ara4N biosynthesis by activating the PhoPQ two-component system [76]. In contrast, the transmembrane protein MgrB acts as a negative regulator of PhoPQ by inhibiting its activity. In *Enterobacter*, resistance to CST is frequently associated with mutations in the *mgrB* gene. Similarly, in *K. pneumoniae*, *mgrB* mutations are one of the most well characterized mechanisms conferring resistance to PMs [76, 77].

### PmrAB

The PmrAB TCS constitutes a crucial adaptive mechanism that allows bacteria to respond to environmental changes, such as elevated concentrations of Al³⁺ and Fe³⁺ concentrations, acidic pH, and antibiotic exposure. The system is encoded by the *pmrCAB* operon, which includes *pmrA* (response regulator), *pmrB* (histidine kinase), and *pmrC* (phosphoethanolamine transferase) [71]. Activation of PmrAB can occur directly or indirectly depending on the stimulus. In direct activation, PmrB senses environmental signals, autophosphorylates, and transfers the phosphate to PmrA. Phosphorylated PmrA then induces transcription of target genes, including *arnBCADTEF* (*pmrHFIJKLM*) and *ugd* (*pmrE*) [71, 78]. Indirect activation is mediated by PmrD, whose expression is regulated by PhoPQ. PmrD stabilizes phosphorylated PmrA by preventing its dephosphorylation. Activated PmrA also promotes transcription of the *pmrCAB* operon and negatively regulates *pmrD*, forming a feedback loop. This interplay between PmrAB and PhoPQ via PmrD has been extensively characterized in *Salmonella* spp. [71].

### Other TCSs regulating the attachment of l-Ara4N

#### CrrAB

**Fig. 6:**
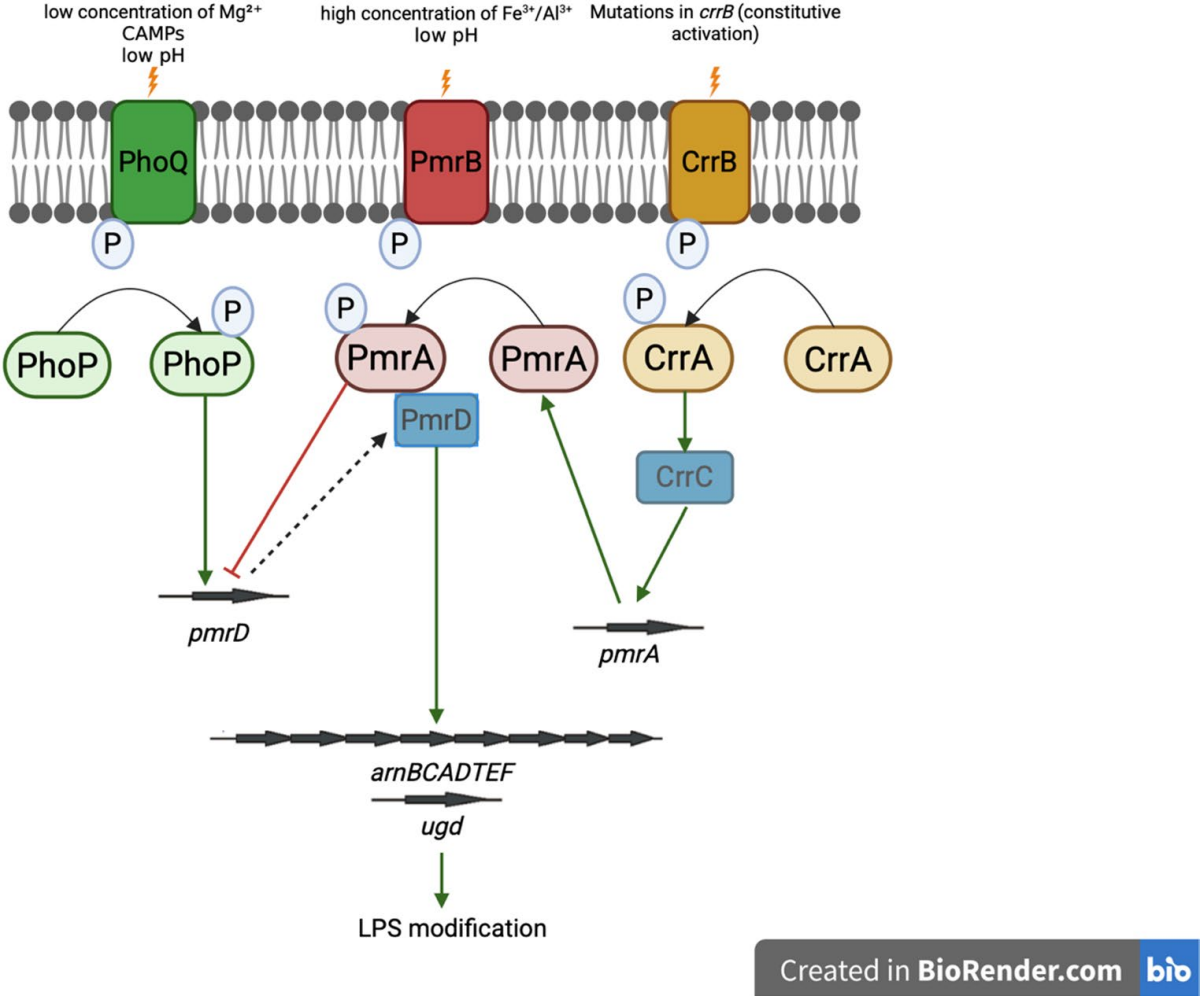
Simplified schematic illustrating the interactions among the two-component systems PhoPQ, PmrAB, and CrrAB involved in the activation of the *arnBCADTEF* operon and the attachment of l-Ara4Nto the LPS structure. Created by the author using BioRender (www.biorender.com) [71, 77, 81]

The regulation of the modification of LPS by the incorporation of l-Ara4N is not exclusively dependent upon the PhoPQ and PmrAB systems. For instance, in *Enterobacter bugandensis*, this process is controlled by both the PhoPQ and CrrAB two-component systems [76]. The CrrAB system consists of the response regulator CrrA and the sensor kinase protein CrrB. In response to environmental signals, CrrB phosphorylates the response regulator CrrA, leading to the expression of the *crrC* gene, which encodes the CrrC protein, and induces *pmrAB* expression. It has been suggested that the CrrC protein mediates the interaction between the PmrAB and CrrAB systems, which implies that CrrAB regulates PmrAB via CrrC. Moreover, mutations in the *crrB* gene increase the expression of CrrC. Transcriptomic and proteomic studies support the role of the CrrAB system in inducing the activity of the *arnBCADTEF* operon [77, 79]. In *K. pneumoniae*, the activation of the *arnBCADTEF* operon is regulated by all three TCSs: CrrAB, PmrAB, and PhoPQ (Fig. 6) [80, 81].

#### ColRS

The ColRS TCS has been identified in *P. aeruginosa* as a contributor to polymyxin resistance. Gutu et al. (2013) demonstrated that resistance in *P. aeruginosa phoQ* mutants (*ΔphoQ*) also depends on ColRS. Deletion of *colR* or *colS* in *ΔphoQ* mutants abolished PMs resistance, although the l-Ara4N modification was still detected. This indicates that ColRS influences l-Ara4N incorporation and the expression of the *arnBCADTEF* operon [82].

### CprS and ParRS

Other relevant TCSs in *P. aeruginosa* include CprRS and ParRS, both of which respond to CAMPs. Unlike *Salmonella*, in which PhoQ detects both low Mg²⁺ and CAMPs via a single mechanism, *P. aeruginosa* employs distinct systems to sense divalent cations (PhoQ/PmrB) and CAMPs (ParS and CprS). Fernández et al. (2012) demonstrated that different CAMPs selectively activate these systems: some peptides exclusively activate CprRS, while others activate both ParRS and CprRS, regardless of peptide concentration [83]. Both systems are activated by PMs, although ParRS plays the dominant role in *P. aeruginosa*. CprRS regulates a narrow set of approximately 15 genes, including *arnBCADTEF* and *pmrAB*, whereas ParRS controls a broader transcriptional response [69]. Moreover, ParRS and CprRS can also be stimulated by host-derived peptides such as the human opioid peptide dynorphin, which enhances virulence factor production and adaptive resistance. It has been proposed that CprRS contributes to host–pathogen interactions via small molecule signaling, although the precise molecular mechanisms remain to be clarified [84].

## Combination therapies with PMs

Polymyxin based combination therapies are being widely explored to overcome resistance and minimize toxicity. Various natural and synthetic compounds have shown synergistic effects with polymyxins (PMB/CST), enhancing antibacterial activity and in some cases reducing adverse effects. Selected examples are summarized below and presented in Table 2.

### Curcumin

Curcumin is a natural polyphenol extracted from the plant *Curcuma longa* and is chemically known as 1,7-bis(4-hydroxy-3-methoxyphenyl)-1,6-heptadiene-3,5-dione. Curcumin has numerous beneficial properties, including anticancer, antioxidant, and anti-inflammatory effects, and most importantly, it exerts bactericidal activity against a wide range of pathogenic bacteria, including antibiotic-resistant strains [85, 86]. Dai et al. (2020) demonstrated a strong in vitro synergistic effect of PMB and curcumin against both Gram-negative bacteria (*Stenotrophomonas maltophilia*, *E.coli*, *A. baumannii*, and *P. aeruginosa*) and Gram-positive bacteria (*S. aureus*). It is hypothesized that this synergism arises because PMs increase the permeability of the bacterial OM, allowing greater intracellular penetration of curcumin. A major advantage of curcumin is its ability to counteract the development of increasing bacterial resistance to PMs by inhibiting efflux pumps [85]. Kaur et al. (2018) demonstrated a strong in vitro synergistic effect between curcumin and colistin against *A. baumannii*. Specifically, curcumin at 100 µM (≈ 36.8 µg/mL) reduced the CST MIC from 2 to 0.5 µg/mL (a fourfold decrease) and inhibited efflux pump activity, thereby enhancing intracellular antibiotic accumulation [86]. Betts et al. (2016) similarly reported 3–10-fold reductions in polymyxin B MICs in various multidrug-resistant Gram-negative isolates when combined with curcumin [87]. Furthermore, another significant benefit of curcumin is its role in mitigating the nephrotoxic and neurotoxic side effects associated with PMs [85, 88]. Further clinical research on polymyxin–curcumin combination therapy is essential, as current evidence on optimal dosing regimens and pharmacokinetic properties remains limited [85].

### Lanthypeptide - Microbisporicin

Microbisporicin is produced by *Microbispora* sp. and belongs to the class I lanthipeptides (bioactive compounds produced by microorganisms). It is characterized by a broad spectrum of activity against bacteria. It is effective against vancomycin-resistant *Enterococcus*, methicillin-resistant *S. aureus*, and Gram-negative strains such as *Moraxella catarrhalis*, *N. meningitidis*, and *Haemophilus influenzae*. Microbisporicin has an additional valuable property: it enhances the activity of PMs antibiotics against Gram-negative bacteria, with observed synergistic effects between the two compounds. Although microbisporicin has not yet been introduced into clinical use, ongoing research aims to facilitate its future application as an adjunct therapy for infections caused by MDR strains [89].

### Netrospine

Netropsin is one of the secondary metabolites produced by *Streptomyces* spp. This compound belongs to the class of pyrrole-amide anticancer drugs, which increase the positive supercoiling of DNA molecules by binding to AT-rich regions in the minor groove. Chung et al. (2016) developed a therapy combining netropsin and PMB to treat infections caused by Gram-negative MDR bacteria and conducted both in vitro studies and in vivo evaluation in a *Galleria mellonella* infection model [90]. They demonstrated that the combined therapy increased the survival rate of *G. mellonella* larvae infected with *A. baumannii* by up to 80% compared with treatment lacking antibiotic. This proposed therapy may be an effective alternative for combating polymyxin resistant bacteria and may minimize the limitations associated with the use of these antibiotics [90].

### Fusidic acid

Fusidic acid (FA) is a natural steroid antibiotic produced by the fungus *Fusidium coccineum*. Its primary action is bacteriostatic, but at higher concentrations, it may exert bactericidal properties. FA has a broad spectrum of activity against Gram-positive bacteria, such as *S. aureus*, whereas most Gram-negative bacteria are resistant to it, with some exceptions among anaerobes. FA also acts as a specific inhibitor of elongation factor G (EF-G), which is involved in peptide translocation during protein synthesis. It is hypothesized that combining FA with other drugs could provide a basis for the development of alternative treatment strategies [91, 92]. Chen et al. (2023) demonstrated that therapy combing FA and PMB enhanced the efficacy of PMB against polymyxin resistant *E. coli* and *K. pneumoniae*, witch a synergistic effect observed across different strains. They also found that the PMB monotherapy was less effective, and that the survival rates in mice were higher when the combined treatment was used [92].

### Cefiderocol

In recent years, there has been increasing interest in the use of cefiderocol (FDC) in combination therapy with PMB, for the treatment of difficult infections caused by XDR *P. aeruginosa*. Romanowski et al. (2025) evaluated the activity of the FDC-PMB combination therapy against *P. aeruginosa* isolates obtained from keratitis, including XDR strains associated with an outbreak of severe infections. Although synergy was not observed for all isolates, the authors excluded the possibility of antagonistic interactions, which is an important consideration for the safety of combination therapy. Additionally, mutants (*baeS*, *fepA*) exhibiting reduced susceptibility to FDC were analyzed; despite these mutations, the strains remained susceptible to treatment with PMB. While clear synergy was not demonstrated across all tested isolates, the absence of antagonism supports the rationale for further preclinical investigations, and potentially future clinical studies [93].

**Table 2:** Synergistic combinations of PMs with other agents against bacteria, including target pathogens, proposed mechanisms of synergy, and experimental outcomes

Combination partner	Target pathogens	Proposed mechanism of synergy	Outcome (in vittro/in vivo)	References
Curcumin	*S. maltophilia*,* E. coli*,* A. baumanii*,* P. aeruginosa*,* S. aureus*	PMs increase OM permeability and enhanced curcumin uptake; inhibition of efflux pumps	Strong in vitro synergy; reduced PMs toxicity	[85, 86, 94, 87, 88]
Microbisporicin – lanthypeptide	MDR Gram-positive and Gram-negative bacteria	Enhances PMs activity by disrupting bacterial cell envelope	Synergy in vitro; potential clinical use under study	[89]
Netrospine	*A. baumannii* (MDR)	DNA binding; PMB increases uptake	Higher survival in *Galleria mellonella* model (up to 80%)	[90]
Fusidic acid Cefiderocol (FDC)	PM-resistant *E. coli*,* K. pneumoniae* XDR *P. aeruginosa*	Inhibition of EF-G; PMB increases uptake Mechanism not demonstrated; no synergy established; no antagonism observed	In vitro synergy; higher mouse survival rates; variable isolate-dependent responses; FDC resistant mutants remained PMB susceptible	[91, 92] [93]

## ArnT transferase inhibitors

With the growing problem of resistance to PMs, increasing attention has been paid to the development of inhibitors that target the bacterial enzymes responsible for the resistance mechanisms. One such enzyme is ArnT transferase, a component of the *arnBCADTEF* operon, which catalyzes the addition of the l-Ara4N moiety to the lipid A portion of LPS. As previously mentioned, the *arnT* gene is highly conserved among various Gram-negative bacterial strains. Therefore, the use of ArnT inhibitors could contribute to the development of novel, more-effective therapeutic strategies for infections caused by polymyxin resistant bacteria, while also mitigating the consequences of rising antimicrobial resistance.

Medicinal plants, and particularly their secondary metabolites, are a rich source of such enzyme inhibitors because they express broad spectrum inhibitory activities against various bacterial targets. One plant-derived compound that has been identified as a potential ArnT inhibitor is ent-beyerane diterpene 1 (BBN149), isolated from *Fabiana densa* var. *ramulosa*. This compound has also been shown to potentiate the activity of CST against resistant strains. It has been suggested that compounds bearing the ent-beyerane scaffold are a promising chemical foundation for the development and optimization of novel ArnT inhibitors aimed at restoring CST efficacy in resistant Gram-negative pathogens e.g. *P. aeruginosa* [95]. However, the use of ent-beyerane diterpenoids may have certain limitations, because these compounds are poorly water soluble, which can negatively affect their biopharmaceutical properties. A possible solution to this problem is the use of liposomes as carriers for these compounds [95, 96].

Pastore et al. (2025) proposed liposomes as carriers for CST and its adjuvants. Isostevic acid (ISA) was selected as a prototype diterpenoid ArnT transferase inhibitor, which also enhances CST activity [97]. This compound was encapsulated together with CST in liposomes, and the resulting formulations were evaluated in vitro for their potential to combat *P. aeruginosa* strains resistant to CST. To assess their applicability, for the treatment of respiratory bacterial infections, the cytocompatibility of the liposomal formulations was tested with a human lung cell line (Calu-3) [97]. Pastore et al. (2025) demonstrated that these liposomal formulations preserved CST activity against various *P. aeruginosa* strains. Their use also allows controlled and potentially targeted drug release, which may help to reduce CST associated toxicity. The codelivery of ent-beyerane-based compounds and CST in liposomal formulations represents a promising strategy to minimize the adverse side effects of CST and facilitate its safe clinical use. This approach may also help to overcome resistance mechanisms based on the modification of LPS through the addition of l-Ara4N [97].

Undoubtedly, ArnT constitutes a promising therapeutic target for overcoming polymyxin resistance; however, there is still a noticeable lack of specific inhibitors directed against this enzyme. The recent elucidation of the ArnT crystal structure provides a crucial framework for the rational design and optimization of novel compounds capable of selectively inhibiting its activity, thereby restoring the efficacy of PMs [98].

Another approach involves targeting the key regulatory pathways of the *arnBCADTEF* operon, specifically the TCSs involved in regulating the mechanisms of PMs resistance. One of the proposed compounds that acts against the PhoPQ system is the well known heat shock protein 90 (Hsp90) inhibitor, radicicol, which inhibits the activity of PhoQ kinase. Cai et al. (2011) identified four chemical compounds as potential inhibitors of PhoQ in *Shigella flexneri*, which inhibit the activity of PhoQ by binding to its cytoplasmic domain. In vitro and in vivo studies have demonstrated that these compounds significantly reduce the virulence of *S.flexneri*. These findings provide new insights into the potential design of therapeutic strategies that target the regulatory pathways responsible for PMs resistance [8, 99].

## Conclusions

Polymyxins, particularly polymyxin B (PMB) and colistin (CST), play crucial roles in the treatment of infections caused by Gram-negative MDR bacteria, such as *P. aeruginosa*, *K. pneumoniae*, *A. baumannii*, and *E. coli*. Despite their strong efficacy against pathogens resistant to many other antibiotics, the use of PMs is limited by their neurotoxicity and nephrotoxicity, necessitating careful dosing and patient monitoring. In recent decades, resistance to PMs has increased significantly, although they are still currently considered last resort antibiotics for infections caused Gram-negative MDR bacteria. This increasing resistance presents a serious clinical challenge and calls for a better understanding of resistance mechanisms, to allow the development of more effective treatment strategies. One key resistance mechanism involves the modification of the LPS structure in the bacterial OM by the addition of l-Ara4N to lipid A. This modification reduces the negative charge on LPS, lowering its affinity for PMs and thus increasing bacterial resistance to these antibiotics.

This LPS modification is regulated by TCSs, such as PmrAB, PhoPQ, CrrAB, CprRS, ParRS, and ColRS, which allow bacteria to adapt to changing environmental conditions, such as low pH, the presence of metal ions, antimicrobial peptides, or oxidative stress. These systems control the expression of the genes responsible for l-Ara4N biosynthesis and attachment, and modulate other bacterial defense mechanisms, including communication and antibiotic response pathways.

With the increasing resistance to PMs, combined with their nephrotoxicity and neurotoxicity, there is growing interest in new therapeutic strategies. Combination therapies that exploit the synergistic effects of PMs and other compounds, such as natural polyphenols (e.g., curcumin), antimicrobial peptides (e.g., microbisporicin), efflux pump inhibitors, and other secondary metabolites (e.g., netropsin or FA) are drawing increasing attention. A novel approach in the fight against Gram-negative MDR bacteria involves the use of ArnT transferase inhibitors. Currently identified inhibitors include compounds with the ent-beyerane skeleton, such as ent-beyerane diterpene 1 (BBN149), which was isolated from *F. densa* var. *ramulosa*. These therapies enhance the efficacy of PMs, reduce the development of resistance, and reduce drug toxicity.

Molecular and transcriptomic studies have also provided deeper insights into the mechanisms regulating resistance, allowing the development of specific inhibitors targeting TCSs or LPS modification pathways, which are promising therapeutic targets. A comprehensive approach to combating Gram-negative infections by integrating an understanding of the resistance mechanisms and novel adjunctive agents is essential to effectively addressing the growing issue of antibiotic resistance.

## Data Availability

No datasets were generated or analysed during the current study.
